# Psorinum: Clinical Reflections

**Published:** 1884-04

**Authors:** Ad. Lippe

**Affiliations:** Philadelphia


### PSORINUM: CLINICAL REFLECTIONS.

AD. LIPPE, M. D., PHILADELPHIA.

Isopathy is a method of curing diseases, first advanced by Lux, which he based on the principle *æqualia æqualibus curantur*. In accordance with this principle, all contagious diseases carry with them in the shape of their infectious matter their curative remedy. Lux drew his deductions from the well-established fact that vaccination proved to be a preventive against small-pox. The vaccine *then* in use successfully was taken from cows suffering from a disease peculiar to them, and this animal poison (a cow-nosode), if used for vaccinating mankind, did certainly *then* diminish the susceptibility to small-pox; or, to express ourselves clearly, fewer were attacked by small-pox after the introduction of vaccination by Jenner. The present mode of vaccination with human scabs or with the artificially produced disease in cows, by a vaccination with human virus, is a "parody on vaccination," and is followed only too often by worse diseases than small-pox. We make these passing remarks only to show the utter fallacy of basing a system of cure on a basis so utterly untenable. A system of cure, furthermore, which only claims to cure infectious diseases can never be regarded as a universal system of cure—such as the Healing Art promulgated by Samuel Hahnemann, and by him called Homœopathy, which is applicable in all forms of non-surgical diseases. If we take in consideration the variety of causes which produce disease, and how easily they may be cured in said manner (labor-saving method), and how little the art of individualization, the great accomplishment of a true healer, comes into play, which art should be an absolute necessity if a permanent and speedy cure is to be accomplished; if we reflect that the homœopathicians have so very often remarked that there seldom or *never* exist two persons who suffer from exactly the same symptoms, but that different persons present *unusual* symptoms, dependent on their own individuality—we are forced to the conclusion that Isopathy is an abortion of Homœopathy and an absolute absurdity as a method of cure.

Among the eccentric followers of Lux we find the early friend and co-laborer of Hahnemann, Dr. Gross; Hahnemann mentions his apostacy in the preface to the *Organon*, where he deals with Isopathy in a foot-note. Dr. Gross had listened to the premature and extravagant promises held out by Lux and did advance into Isopathy and became an eccentric defender of that absurdity. Upon mature reflection he returned to pure and simple Homœopathy, and the bubble burst. Isopathy was for the time abandoned.

The isopathists of the Lux order were not the first men who introduced all sorts of nastiness into the Pharmacopœa. Paulini wrote in 1687 his Pharmacopœa, which comprised excrements of all kinds, urine, placenta, worms, all sorts and parts of animals well powdered (triturated?), the deer horn of the narwhal was to cure all cases of poisoning and every contagion, the effusion of the viper was a specific for all debilities, and sterility was to be cured with the pulverized testicles of a ram.

Hahnemann gave in the *Chronic Diseases* his reasons for not including among the antipsoric remedies the nosode *Psorinum*, and his very valid reason was that Psorinum was not sufficiently proved. Not only a proving of a drug was in those days considered necessary before a new remedy could be incorporated into our materia medica, but it was considered absolutely necessary to have the provings verified by the clinical experiment, and therefore Psorinum had—to wait for verifications.

If Isopathy was correct, in the propositions made by Lux of old, and also by the reviver of Lux's apostacy, the Psorinum would cure all and every case of the "itch." The fact is that as far as we know Psorinum has rarely ever cured a case of the "itch" proper, *scabies sicca*. The clinical experiments of this now well-proved remedy show very singular cures when it was administered under the exclusive law of the similars. We find, for instance, a case related in *Rueckert's Homœopathic Therapia*.

Psorinum 30, one globule dissolved in 4 oz. of water for three days, one tablespoonful a day, relieved a case of religious melancholy in an epileptic patient.

Psorinum 30, two globules in seven doses, one dose given every fortnight, cured almost completely a scrofulous inflammation of the eyelids. The eyelids were covered with thick crusts; at the same time almost the whole body was covered with a bran-like tetter.

Psorinum 30, two globules repeated every eight days, cured in a boy inguinal hernia reaching down to the testicles. The hernial sack, in consequence of previous inflammations, contained a large quantity of water—complete cure (Achiv. XIV, 2, p. 136).

Psorinum appears to be one of the remedies in chronic constipation (Allg. Hom. Ztg., 2, p. 69).

Psorinum 30, two globules, two doses eight days apart, cured a case of hydrocele caused by repeated inflammation in consequence of pressure from a truss.

Psorinum seems to be effective in certain forms of dry coryza with stoppage of the nose (Allg. Hom. Ztg., II, p. 69).

Psorinum 10 cured a dry cough with dyspnœa and a pain in the chest as if it were raw and scratched (Arch. XII, 2, p. 90).

Psorinum 2 was useful in not far advanced phthisis pulmonalis purulenta (Allg. Hom. Ztg. V., p. 107).

Psorinum in repeated dose prevented the suppuration of tubercles in the lungs. Symptoms: Dull pressure extending from the right side all over the chest; aggravated by bending forward; mostly dry cough with expectoration of small, lumpy masses; very much exhausted by talking; the voice is not hoarse but full; much tired from preaching; chest contracted; shoulders standing forward.

Psorinum 30, three pellets, a dose once a month, removed chronic rheumatism in the limbs with a dry eruption on the wrists.

Psorinum 30, repeated three times, removed almost entirely a pain in the knee caused by a fall a year ago.

Psorinum 30, two doses, cured in a child an offensive smelling crusty eruption extending over the whole face which for three months had completely closed the eyes (Arch. XIV, 3, p. 132).

Psorinum 30, three doses, a previous tetter on the arm with small, millet-like eruption exuding a yellow fluid. The eruption itches intensely in the heat.

Psorinum 30, three doses every month, once cured a dry tetter on the wrists with rheumatism in the limbs.

Psorinum cured in three days a copper-colored eruption on (the top of) the hand.

Psorinum, two doses, cured in a month a moist scab behind the ears with dry tetter on the back of the head, on both cheeks extending upward to the eyes and downward to the corners of the mouth, reddish, very closely packed, milletseed-like itching, dry pimples, with frequent loose stools, in a child one and a half years old.

Psorinum 30, one dose cured in a month the eruption in the face of a child. The whole face was covered by a crust, lips and eyelids were swollen, aversion to light, large, moistening spots on the head and behind the ears (Allg. Hom. Ztg. IV, p. 14).

Psorinum 30, two doses in a fortnight cured large condylomata located and extending around the edge of the prepuce, moist, itching, and at times burning, at the same time involuntary urinary secretion at night and frequent micturition during the day, small quantities being emitted, with burning in the condylomata and the urethra, ulcerated lips and dry tetter in the bends of the knee (Allg. Hom. Ztg. IV, p. 14).

Psorinum, two doses, cured a malignant boil. Symptoms: On the hand a cone-shaped scab in the size of a quarter of a dollar, on a base as large again, bluish red and strongly demarcated, where the scab extends over the ring there is another white moist ring which forms a new scab. The whole causes much tension and burning (Allg. Hom. Ztg. III, p. 117).

Psorinum is recommended by Gutt. Archiv. 14, 2, 137, as a possible successful remedy in hydrocele; he recommends it referring to a cure of a case of hernia with accumulation of water in the hernial sack. Later cases of hydrocele cured with Psorinum have been reported.

Psorinum has often cured the consequences of itch suppressed by the use of sulphur ointment.

Psorinum has some very prominent guiding symptoms, as, for instance, dyspnœa, worse when sitting up and relieved by lying down; congestion to the head after dinner; great despondency predominates.

This collection of clinical reports, made in former years, shows that while Psorinum is by no means a specific for the "itch," the results of careful provings have enabled the healer to make good use of it in a great variety of ailments. It is obvious that the assertions of the isopathists are a fallacy; it is obvious that in former years the 30*th potency* did cure the sick when the remedy was homœopathic to the case; it is obvious that its homœopathicity depended on the *similarity* of the symptoms of the patient with those observed on the provers; it is also obvious that the symptoms of the itch miasm do not constitute a proving, as has been claimed by some, who also claim that Syphilinum and other nosodes have been sufficiently proved in the sufferings of those infected, that no other provings were necessary, *but* instead of an old-fashioned proving they must be "highly potentized" and thereby become reliable, truly homœopathic remedies. Where is "the logic" of these eccentric men? Jenner's small-pox preventive is the corner-stone on which is built the new labor-saving healing art. A poor foundation at best for anything; but if, for mere argument's sake, we admit the correctness of Jenner's theory, what relation can morbific products of sick men have to the morbific product of a lower animal? Again, for argument's sake, suppose he is exempt, what comfort can thereby come to the advocates of modern Luxism with a variation. Jenner's remedy is the morbific product of a cow disease transferred on man to protect him against small-pox. The modern advocates of Luxism with a variation claim that the morbific product of a disease taken from one human being *if highly potentized* (that is, the variation) will cure the same disease in others; and that is boldly claimed to be "Homœopathy." Since when does Homœopathy treat diseases—we mean forms of diseases?

Hahnemann tells us in paragraph 153, of his *Organon:* "The search for a homœopathic specific remedy consists in the *comparison* of the totality of the symptoms of the natural disease with the lists of symptoms of our tested drugs, among which a morbific potency is to be found corresponding in similitude with the disease to be cured. In making this comparison, the more *prominent, uncommon,* and *peculiar* (characteristic) features of the case are especially, and almost exclusively, considered and noted; for these in particular should bear the closest similitude to the symptoms of the desired medicine, if that is to accomplish the cure."

What we want are "provings"—*genuine provings,* not symptoms accidentally cured by the hap-hazard administration of an unproved *but* highly potentized remedy to be set down as symptoms absolutely obtained by proving the drug on the healthy, that is laborious, and this is exactly where the shoe pinches. We hope we have shown the absurdity of Luxism with and without modern (ingenious?) variations, and we have chosen to give clinical reflections on Psorinum, one of the well-proved nosodes, to show the proper and only practicable method to "advance." Sober and logical reflections will convince every sensible man in what manner we may advance and develop the healing art.

